# Polysaccharide-based liquid storage and transport media for non-refrigerated preservation of bacterial pathogens

**DOI:** 10.1371/journal.pone.0221831

**Published:** 2019-09-06

**Authors:** Janine R. Hutchison, Shelby M. Brooks, Zachary C. Kennedy, Timothy R. Pope, Brooke L. Deatherage Kaiser, Kristin D. Victry, Cynthia L. Warner, Kristie L. Oxford, Kristin M. Omberg, Marvin G. Warner

**Affiliations:** 1 Chemical and Biological Signature Sciences Group, National Security Directorate, Pacific Northwest National Laboratory, Richland, WA, United States of America; 2 Subsurface Science and Technology Group, Energy and Environment Directorate, Pacific Northwest National Laboratory, Richland, WA, United States of America; 3 Integrated Omics, Earth and Biological Sciences Directorate, Pacific Northwest National Laboratory, Richland, WA, United States of America; Instituto Butantan, BRAZIL

## Abstract

The preservation of biological samples for an extended time period of days to weeks after initial collection is important for the identification, screening, and characterization of bacterial pathogens. Traditionally, preservation relies on cold-chain infrastructure; however, in many situations this is impractical or not possible. Thus, our goal was to develop alternative bacterial sample preservation and transport media that are effective without refrigeration or external instrumentation. The viability, nucleic acid stability, and protein stability of *Bacillus anthracis* Sterne 34F2, *Francisella novicida* U112, *Staphylococcus aureus* ATCC 43300, and *Yersinia pestis* KIM D27 (pgm-) was assessed for up to 28 days. Xanthan gum (XG) prepared in PBS with L-cysteine maintained more viable *F*. *novicida* U112 cells at elevated temperature (40°C) compared to commercial reagents and buffers. Viability was maintained for all four bacteria in XG with 0.9 mM L-cysteine across a temperature range of 22–40°C. Interestingly, increasing the concentration to 9 mM L-cysteine resulted in the rapid death of *S*. *aureus*. This could be advantageous when collecting samples in the built environment where there is the potential for *Staphylococcus* collection and stabilization rather than other organisms of interest. *F*. *novicida* and *S*. *aureus* DNA were stable for up to 45 days upon storage at 22°C or 40°C, and direct analysis by real-time qPCR, without DNA extraction, was possible in the XG formulations. XG was not compatible with proteomic analysis via LC-MS/MS due to the high amount of residual *Xanthomonas campestris* proteins present in XG. Our results demonstrate that polysaccharide-based formulations, specifically XG with L-cysteine, maintain bacterial viability and nucleic acid integrity for an array of both Gram-negative and Gram-positive bacteria across ambient and elevated temperatures.

## Introduction

The ability to preserve microbial specimens obtained from various sources is valuable to clinicians, researchers, and industry in numerous areas such as agriculture, food, and public health. Specifically, the ability to accurately detect and screen for bacterial pathogens, whether naturally occurring or intentionally or accidentally released, in an environmental or non-clinical setting is of critical importance [1]. In these circumstances, it is imperative to stabilize the viable sample containing the pathogenic material after collection and subsequently avoid overgrowth and/or an invasion of contaminants that may deteriorate the sample. Furthermore, it is important that the stabilization system does not complicate or interfere with downstream characterization, detection, or analytical methods focused on identifying critical biomarkers such as DNA, peptides, proteins, and small molecules. In a remote field collection setting, it is also impractical to rely on traditional preservation methodology employing complicated, costly, or bulky instrumentation such as ultra-low temperature (i.e. −80°C) freezers, spray-dryers, freeze-dryers, etc. Therefore, there is an impetus to employ simple biological stabilization tools and protocols that are effective at preserving field collections at indoor/outdoor ambient temperatures over periods of weeks or even months or in conditions mimicking those encountered during transport.

Transport of bacterial samples for offsite laboratory analysis poses multiple problems. Current stabilization methods are time and labor intensive and may require environmental controls (such as temperature) that are not available in remote locations. Silvestri et al. has summarized the challenges of collecting and interpreting field data, including surface sampling device (swab) collection, detection of pathogens in environmental matrices, as well as sample transport challenges for *B*. *anthracis* spore/vegetative recovery [2]. Biological samples may degrade during the shipment time and lose viability or degrade and denature nucleic acids, which makes detection of the organism difficult by polymerase chain reaction (PCR) or immunoassay techniques. The transport media ideally must also be able to maintain a relatively constant cell concentration reflecting the initial sampling concentration and avoid overgrowth of non-target organisms. The shipment of samples on dry ice or in refrigerated conditions (4°C) may help maintain viability and protect nucleic acid degradation to some extent, but it can be difficult to maintain a constant cold chain in remote locations.

Several studies have been conducted to evaluate the viability and stabilization of bacterial pathogens including organisms such as *Bacillus anthracis* and *Yersinia pestis* after storage in commercial-off-the-shelf (COTS) solutions at or above 4°C [3–6]. Typically, high cell concentrations either directly spiked into solution (at 10^6^ colony forming unit (CFU)/mL) or onto swabs (at 10^8^–10^9^ CFU/mL) have been examined after storage in various COTS solutions at a range of temperatures and their viability assessed by plate counting. Notably, Sanicult^TM^ solution (Thermo Fisher Scientific) was found to maintain viable *B*. *anthracis* cells over a period of 14 days after storage at 40°C [5]. Viable *Y*. *pestis* cells were found when stored at 4°C for 72 hours in either neutralizing buffer or phosphate buffered saline (PBS) with surfactant [7]. These studies provided insight towards understanding the basic stability of pathogenic samples in liquid media without cold chain storage. However, there is further need for understanding how the transport media composition influences bacterial preservation, particularly in non-ideal conditions (i.e. ambient temperatures), at lower cell concentrations, and over longer storage times. Generally, the range of materials currently employed as stabilization media in liquid form is limited. Liquid formulations used to date have been primarily composed of simple salted buffers (i.e. PBS, Butterfield’s phosphate) or biologically derived enrichment media with abundant nitrogen, carbon, vitamin, and mineral sources (i.e. peptone, broths, skim milk). For example, the commercial Sanicult product contains buffered peptone water (20.0 g/L) according to the manufacturer. Despite the success of stabilizing certain organisms, commonly used media may not be optimized for other important classes of pathogenic bacteria. For example, *Francisella* spp. typically require small molecules such as cysteine for growth and enhanced culture recovery. Furthermore, it is difficult to establish structure–preservation ability relationships for these biologically-derived complex peptone mixtures and start to rationally design more effective formulations.

Soluble polymeric materials were envisioned as alternative formulation matrix materials that may be able to interact with bacteria cells, provided basic supplementation requirements are met (i.e. pH, salts), and act as preservatives. Specifically, dissolved polysaccharide-based polymer substrates were thought to have potential as room temperature preservatives. Previously, natural biopolymers with glycoprotein and polysaccharide compositions including acacia gum and pullulan gum were found to be effective stabilizers of Gram-negative (*Escherichia coli*) and sporulating Gram-positive (*Bacillus subtilis*) bacteria at or above room temperature when desiccated [8]. In addition, polysaccharide-based (xanthan gum, XG) dehydrated gels were effective for immobilizing a variety of bacteria, fungal spores, and yeast for extended time periods at 28°C [9]. In these cases, the microorganism was immobilized and preserved only after a desiccation step. Inspired by these studies, we wished to explore the room temperature preservation of bacteria pathogens in liquid formulations comprised of dissolved polysaccharide polymers. As polysaccharide polymers are typically viscosity-enhancing, the aim was to keep the concentration low enough to ensure the formulations remain as solutions (i.e. do not undergo significant gelation) but high enough to induce stabilization. Practically speaking, retention of a moderate to low viscosity in a formulation enables simple manipulation via pipet, which simplifies quantification experiments such as viable plate counting and is expected to improve compatibility when directly used in downstream detection approaches.

Herein, the effectiveness of polysaccharide-based liquid formulations for the preservation of viable bacteria and characteristic nucleic acid and protein signatures at up to 45 days is described. The bacterial agents selected for this study were as follows: *Bacillus anthracis* Sterne, a Gram-positive spore-former, *Staphylococcus aureus*, a Gram-positive non-spore forming organism, and Gram-negative organisms *Francisella novicida* U112 and *Yersinia pestis* KIM D27. After the organisms were stored in various solutions, the effectiveness of the storage formulation was evaluated using viable plate counts. Nucleic acid stability was tested by performing real-time PCR and monitoring C_T_ values over time. A range of incubation temperatures (22, 30, 35, and 40°C) was tested to mimic conditions encountered during the transport process from field collection to laboratory analysis [5]. Various COTS solutions were evaluated and compared to the specialized media formulations.

## Materials and methods

### Reagents and solutions

Aqueous solutions were prepared in either Phosphate Buffered Saline (PBS, Gibco without calcium and magnesium) or Remel^™^ Butterfield’s Phosphate Buffer, Thermo Fisher Scientific (Waltham, MA). A 0.5% (weight/volume in buffer) xanthan gum (XG) solution (from *Xanthomonas campestris*), Sigma-Aldrich (St. Louis, MO), was prepared one day prior to conducting an experiment. The mixture was shaken at 35°C typically for 3 h to initiate the mixing process and then sterilized by autoclaving. After sterilization, the mixture was homogeneous and could be stored until use at room temperature. Additives such as L-cysteine (L-cysteine, ≥ 98.5% BioUltra), and sodium thioglycolate were obtained from Sigma-Aldrich (St. Louis, MO). Peptone buffered water (PBW, 5 g/mL) was purchased from Neogen (Lansing, MI). Sanicult Medium was purchased from Thermo Fisher Scientific (Waltham, MA).

### Viability assay

The following bacterial strains were used to evaluate viability, protein, and nucleic acid stability: *Bacillus anthracis* Sterne 34F2, *Francisella novicida* U112, *Staphylococcus aureus* ATCC 43300, and *Yersenia pestis* KIM D27 (pgm-). Sixteen-hour cultures were grown in Tryptic Soy Broth (TSB), Becton Dickinson (Franklin Lakes, NJ), at 37 or 30°C (*Y*. *pestis*) with shaking at 200 rpm. Bacterial cultures were then pelleted by centrifugation (14,000 × *g* for 10 minutes) and washed three times in Butterfield’s buffer (Remel). Washed cultures were then diluted to the target cell concentration in Butterfield’s buffer prior to spiking into the stabilization formulations. Formulations were then stored in the dark at various temperatures (22, 30, 35, or 40°C) up to 45 days. Viability was determined through serial dilutions and plating in triplicate on to Tryptic Soy Agar (TSA) Becton Dickinson (Franklin Lakes, NJ) at pre-determined time points. Plates were incubated at the appropriate temperature for each organism. Bacterial viability is reported as viable log CFU/mL. Log unit change was calculated by dividing the viable log CFU/mL by the starting log CFU/mL, and the results are reported as the average ± standard deviation of the CFU/mL. The number of biological and sample replicates is denoted throughout the results and discussion for each experiment.

### Sampling study

Hutchison et al.[10] previously described the preparation of the 2 inch × 2 inch stainless steel coupons. Bacterial cells were washed and diluted in Butterfield’s buffer to the target concentration prior to deposition onto the stainless steel or glass coupon surfaces as ten, 10 μL drops. The starting concentration was determined using serial dilutions and viable plate counts. Inoculated coupons were air dried for 2 hours in a biosafety cabinet. A macrofoam swab (#25–1607 1PF SC; Puritan, Guilford, ME) was used to collect the bacterial cells from the surfaces using the CDC protocol for surface sampling *B*. *anthracis* spores from smooth, nonporous surfaces [11]. After the sample was collected, the swab was transferred to a 5 mL tube containing 3 mL stabilization formulation. The stick was removed from the swab head and the sample was stored at 22°C. At various time points (including immediately after collection), bacterial cells were eluted off the swab head via vortexing at 2,000 rpm for 2 minutes using a Fisherbrand^™^ digital Multitube vortexer, and the supernatant was plated on to TSA plates. Plates were incubated at the appropriate temperature for each strain.

### Nucleic acid analysis

DNA standards were generated from the genomic DNA of each organism using the MasterPure^™^ DNA Purification Kit (Epicentre Biotechnologies, Inc., Madison, WI). Nucleic acid concentration was measured using a nanodrop and was then diluted in TE buffer (Ambion, Thermo Fisher Scientific [Waltham, MA]) to a working concentration of 2 ng/μL. The stock standard was diluted serially 1:10 to generate a five-point standard curve samples.

At pre-determined time points, 200 μL of bacterial cells in the stabilization formulation was collected and stored at −20°C. After all time points had been collected, real-time PCR was performed using an ABI 7500 instrument with standard fast cycling conditions. Each sample was run in triplicate as a 20 μL reaction containing the following: 10 μL TaqMan Fast Universal PCR Master Mix (2x) (4367846 Thermo Fisher Scientific [Waltham, MA]), 5 μL of template (experimental sample, genomic standard, or TE), 4 μL nuclease free water, and 1 μL of primer probe mixture. Direct PCR analysis of experimental samples was conducted after heat lysis directly in the well without any DNA extraction steps. Primers and probes were purchased from Integrated DNA Technologies (Coralville, IA) as a PrimeTime XL qPCR Assay and a final concentration for the reaction was 1×. Primer sequences are indicated in Table 1, *S*. *aureus* primers were designed targeting SodA, *F*. *novicida* primers have been reported previously [12].

**Table 1:** Primers and probes used for DNA stability assessment.

Strain	Primer name	Sequence (5′ – 3′)
***F*. *novicida* U112**	Ipn_F	CGC AGG TTT AGC GAG CTG TT
	Ipn_R	GCA GCT TGC TCA GTA GTA GCT GTC T
	Ipn_P	/56-FAM/CA TCA TCA G/ZEN/A GCC ACC TAA CCC TA/3IABkFQ/
***S*. *aureus*** **ATCC 43300**	Sa_sodA_F	GCT GCA GTA GAA GGT ACA GAT T
	Sa_sodA_R	GTT TAA ATG TCC ACC GCC ATT AT
	Sa_sodA_P	56-FAM/CC AGC TAA C/Zen/A TCC AAA CTG CTG TAC GT/3IABkFQ/

### Proteomic analysis

#### Peptide sample preparation

Chemicals and reagents were purchased as reagent grade from commercial suppliers, typically Sigma-Aldrich (St. Louis, MO). Bacterial cells from biostabilization samples were pelleted by centrifugation for 30 minutes at 16,000 × *g* at specific time points. Pellets were washed one time with PBS and then stored at −70°C until sample preparation. Proteins were extracted and peptides were purified as previously described by Deatherage Kaiser et al[13]. Briefly, frozen cell pellets were thawed and then resuspended in 0.5 mL of 20% w/v trichloroacetic acid (TCA). The samples were then incubated for 24 hours at −20°C. Samples were thawed and centrifuged, supernatant was discarded, and the remaining pellet was washed twice with 0.2 mL of cold acetone. The pellet was dried and then resuspended in 0.1 mL of a denaturation solution containing 6 M urea and 14.3 mM β-mercaptoethanol at 60°C with vigorous shaking. After one hour, 900 μL of 50 mM ammonium bicarbonate was added to the sample to dilute urea to < 1 M, and Promega Gold Trypsin (V5280 Promega [Madison, WI]) was added to a final ratio of 1:100–1:20. Samples were incubated overnight (18 h) at 37°C with shaking (150 rpm). Peptides were cleaned using C18 solid phase extraction columns (Phenomenex, Torrance, CA) according to the manufacturer protocol. Cleaned peptide samples were brought to near dryness in a speed vacuum and then resuspended in 0.1% formic acid. Peptide concentration was determined using the bicinchoninic acid assay (Pierce, Appleton, WI); all samples were adjusted to 0.2 μg/μL prior to LC-MS/MS analysis.

#### Liquid chromatography mass spectrometry

Liquid chromatography separation was performed using an Agilent 1200 HPLC with a 40 cm long 0.15 mm ID fused silica column packed with Jupiter 5 μm C-18 resin. Mobile Phase A (5% acetonitrile, 0.1% formic acid in nano-pure H_2_O) and Mobile Phase B (95% acetonitrile, 0.1% formic acid in nano-pure H_2_O) were adjusted to establish a reversed phase gradient transitioning from 0% Mobile Phase B to 45% Mobile Phase B over the course of 60 min at a flow rate of 2 μL per minute. A wash and a regeneration step were performed following this step. Eluate from the HPLC was directly ionized and transferred into the gas phase with an electrospray emitter (operated at 3.5 kV relative to the mass spectrometer interface). The ion transfer tube on the Orbitrap XL system (Thermo Electron, Thousand Oaks, CA) was maintained at 200°C and 200 V, with an ion injection time set for automatic gain control and a maximum injection time of 200 ms for 5 × 10^7^ charges in the linear ion trap. Dynamic parent ion selection was used, in which the five most abundant ions were selected for MS/MS using a 3 m/z window. Each sample was analyzed in technical triplicate for these studies.

#### Data analysis

LC-MS/MS raw data files were searched against matching proteome sequences using the search algorithm MSGFPlus [14]. The following sequences were used: *Francisella tularensis* subsp. *novicida* U112 (NCBI Accession PRJNA236529) and *Xanthomonas campestris* ATCC 33913 (NCBI Accession GCF_000007145.1).PRJNA448646). MSGFPlus output was filtered to a false discovery rate of 0.003% using the MSGFPlus SpecProb score cut-off of ≥ 1 × 10^−10^. The data were further filtered to remove any spectral counts equal to 1 and to remove any proteins observed in fewer than 6 out of 72 datasets. All protein identifications are provided as a crosstab spreadsheet in S1 Table. Data were visualized using the program InfernoRDN [15, 16].

## Results and discussion

In this study, matrix materials were designed to act as transport and storage formulations capable of maintaining bacterial viability as well as nucleic acid and protein integrity without cold-storage. Prerequisite physical properties for matrix polymeric materials ideally included low vapor pressures, low chemical reactivity, high thermal and chemical stabilities, easily transferred via a pipette, minimal toxicity, and compatibility with PCR and LC-MS/MS analysis. The primary aim of this formulation development is to maintain more viable bacterial cells than COTS products. In addition, an ideal stabilization formulation would be nutritionally limited to prevent target and non-target bacterial or fungal overgrowth. Initially, *Francisella novicida* U112 was selected as the model organism due to unique nutritional requirements and as a surrogate for *Francisella tularensis* [17].

### Initial screening of commercial media and xanthan gum formulations for viability maintenance of *F*. *novicida* U112

Five baseline buffers and commercial storage media were screened for the preservation of *F*. *novicida* U112 over an initial period of 4 days at both 22 and 40°C (Table 2) based on previous studies referenced in the introduction. PBS, 0.1% peptone, Sanicult, and 5 g/L peptone buffered water (PBW) maintained *F*. *novicida* viability with a 0.5 to 2-log reduction over four days when stored at 22°C. Storage in Butterfield’s buffer resulted in a 3-log change reduction of *F*. *novicida* over four days. PBW was the only COTS material to maintain *F*. *novicida* viability at 40°C after 4 days of incubation, with a ~2 log reduction in viable cells.

**Table 2:** Maintenance of *F*. *novicida* U112 viability in commercial buffers at two temperatures.

		Bacterial Recovery (log CFU/mL)				
Buffer Medium	Temperature	Day 1	Day 2	Day 3	Day 4	Log-unit change
**0.1% Peptone**	22°C	8.2 × 10^6^	2.9 × 10^6^	7.9 × 10^5^	9.8 × 10^4^	–2.0
	40°C	1.8 × 10^3^	0	0	0	–7.0
**Butterfield's Buffer**	22°C	3.5 × 10^6^	5.8 × 10^5^	1.2 × 10^5^	9.2 × 10^3^	–3.1
	40°C	0	0	0	0	–7.0
**PBS**	22°C	5.0 × 10^6^	3.1 × 10^6^	1.2 × 10^6^	2.7 × 10^5^	–1.6
	40°C	2.0 × 10^6^	0	0	0	–7.0
**Sanicult**	22°C	8.7 × 10^5^	5.3 × 10^5^	3.2 × 10^5^	2.7 × 10^5^	–1.6
	40°C	3.0 × 10^6^	5.7 × 10^4^	8.0 × 10^1^	0	–7.0
**5 g/L Peptone buffered water**	22°C	Not collected	Not collected	Not collected	2.6 × 10^5^	–0.5
	40°C	Not collected	Not collected	Not collected	4.3 × 10^3^	–2.3

**Fig 1:**
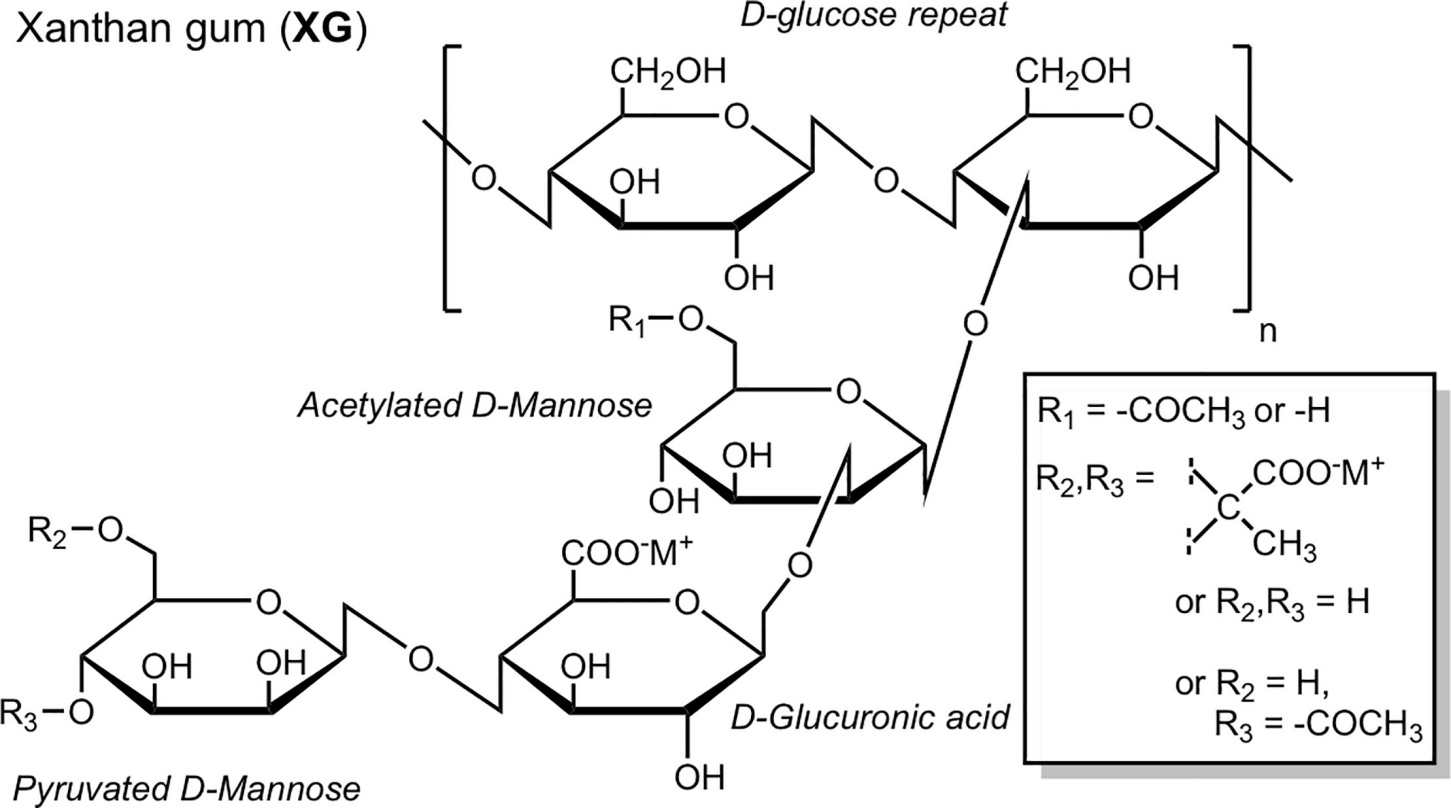
Structure of xanthan gum (XG) with sub-structural components denoted. For XG, a majority of R1 groups on the inner mannose residue are 6-acetylated and an estimated 40% of the terminal mannose residues are 4,6-pyruvated (R2, R3).

Beyond COTS formulations, natural biopolymers or synthetic variants with polysaccharide backbone structures were identified as materials that satisfy the desired physical prerequisites listed above. As described in the introduction, polysaccharides have previously received attention for their ability to serve as a platform capable of entrapping, immobilizing, or interacting favorably with various biological materials. Preliminary screening identified xanthan gum (XG), a biopolymer produced by *Xanthomonas campestris*, as a potentially favorable material capable of stabilizing bacteria at a range of temperatures in relatively low concentration when dissolved in simple buffers (Fig 1). The effectiveness of this simple XG formulation(s) was first compared to COTS reagents and buffer controls.

XG was dissolved in various aqueous diluents (deionized water, Butterfield’s buffer, and PBS buffer) at 0.5% (w/v). The measured viscosity of 0.5% XG (w/v in PBS) remained low at this concentration with a value of 5.1 cP at 22°C. For comparison, the viscosity of a pure H_2_O sample was 0.9 cP at 22°C. Each formulation was used to maintain viability of *F*. *novicida* U112 over 14 days at 22 and 40°C (Table 3). Upon storage in XG in deionized water, viable cells were only recovered after 4 days of storage at 22°C, and no viable cells were recovered at any time point at 40°C in this formulation. Similar results were observed for samples stored in XG in Butterfield’s buffer (pH 7.2) with viable cells present up to day 7. Preparation of XG in PBS (pH 7.4) generated the most successful stabilization formulation at 22°C. A one log increase in total cells was observed across the time points. PBS contains sodium chloride, whereas Butterfield’s buffer does not; these data suggest that this difference improves the stabilization of *F*. *novicida* at this temperature perhaps through osmotic balance. Both commercial products tested did maintain viable cells at 22°C, with PBW performing slightly better than Sanicult. Overall, there was a greater loss of bacterial cells compared to XG in PBS; thus, XG in PBS was selected for additional testing and optimization. It is also important to note that both commercial products contain nutrient dense components such as peptones that may promote overgrowth of non-target bacteria and fungi in environmental samples.

**Table 3:** Bacterial recovery of *F*. *novicida* in XG-based formulations.

		Bacterial Recovery (log CFU/mL)			
Formulation	Temperature	Day 4	Day 7	Day 14	Log-unit change
**0.5% XG in H**_**2**_**O**	22°C	1.0 × 10^2^	0	0	–5.9
	40°C	0	0	0	–5.9
**0.5% XG in Butterfield’s Buffer**	22°C	2.4 × 10^4^	3.0 × 10^3^	0	–5.9
	40°C	0	0	0	–5.9
**0.5% XG in PBS buffer**	22°C	1.2 ×10^5^	1.2 × 10^6^	1.7 × 10^6^	+1.0
	40°C	0	0	0	–6.0
**5 g/L Peptone buffered water**	22°C	2.6 × 10^5^	7.5 × 10^3^	8.8 × 10^3^	–2.0
	40°C	4.3 × 10^3^	1.7 × 10^4^	0	–5.9
**Sanicult**	22°C	2.6 × 10^4^	1.2 × 10^4^	3.6 × 10^2^	–3.4
	40°C	0	0	0	–5.9

### Optimization of XG-based formulations with sulfur-containing small molecules for the preservation of *F*. *novicida*

**Fig 2:**
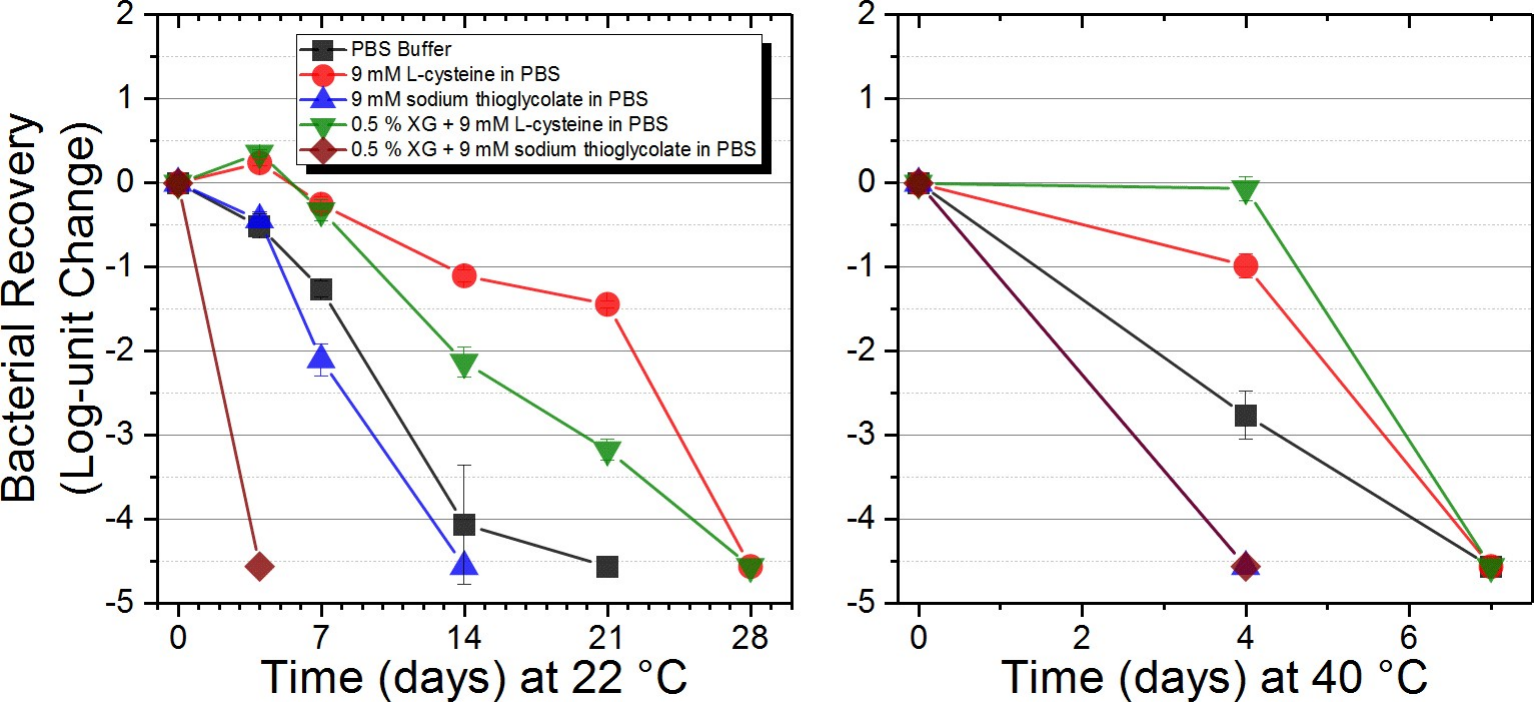
Screening sulfur sources at varying concentration to maintain *F*. *novicida* viability. XG and reference buffers were supplemented with two sulfur sources at varying concentrations. Viability of *F*. *novicida* in test matrices were spiked with ~10^4^ CFU/mL and stored at 22 and 40°C for 28 days. Data are the average of one biological replicate and two sample replicates plated in triplicate (n = 6 measurements).

Improved growth and culture stability of *Francisella spp*. (*tularensis*) has been reported with the addition of a sulfur source, specifically L-cysteine [17, 18]. Thus, we hypothesized that the addition of an exogenous sulfur source may improve the stabilization of *F*. *novicida* under the test conditions. Experiments were performed to determine if sulfur-containing small molecules affect *F*. *novicida* stabilization. L-cysteine (9 mM) and sodium thioglycolate (9 mM) were added to both PBS buffer and 0.5% XG in PBS. *F*. *novicida* viability was assessed over 28 days at both 22 and 40°C (Fig 2). The concentration of the sulfur sources was based off of the concentration of the sulfur-source in other biological buffers such as Amies or Stuart media. PBS was used as a baseline control. At 22°C, PBS buffer supplemented with L-cysteine at both concentrations maintained the highest viability at 21 days storage with a ~1–1.5 log reduction; however, no viable cells were detected at 28 days. A combination of XG with L-cysteine (9 mM) maintained viability for 21 days with a ~3 log reduction, but again no cells were found viable at 28 days. Notably, sodium thioglycolate (9 mM) in PBS with and without XG decreased the stabilization times and viable cell counts to lower than the PBS-only control solution. Multiple L-cysteine-containing formulations afforded the best stabilization at 40°C and when compared to commercial materials. Little to no loss of *F*. *novicida* viability was observed when stored in XG with L-cysteine (9 mM) at 4 days. These results suggest that formulations supplemented with L-cysteine specifically enhance the stability of *F*. *novicida* and are particularly advantageous for storage at high temperatures. Note that the starting cell counts used in these studies are relatively low (~10^4^–10^5^ CFU/mL). The lower cell count used throughout this optimization process is expected to be more challenging for stabilization as compared to previous studies on stabilizing pathogenic bacteria that employ cell counts ranging from 10^6^–10^9^ CFU/mL [4, 5].

### Optimization of L-cysteine concentration for viability maintenance of *B*. *anthracis*, *F*. *novicida*, *S*. *aureus*, and *Y*. *pestis* over time

**Fig 3:**
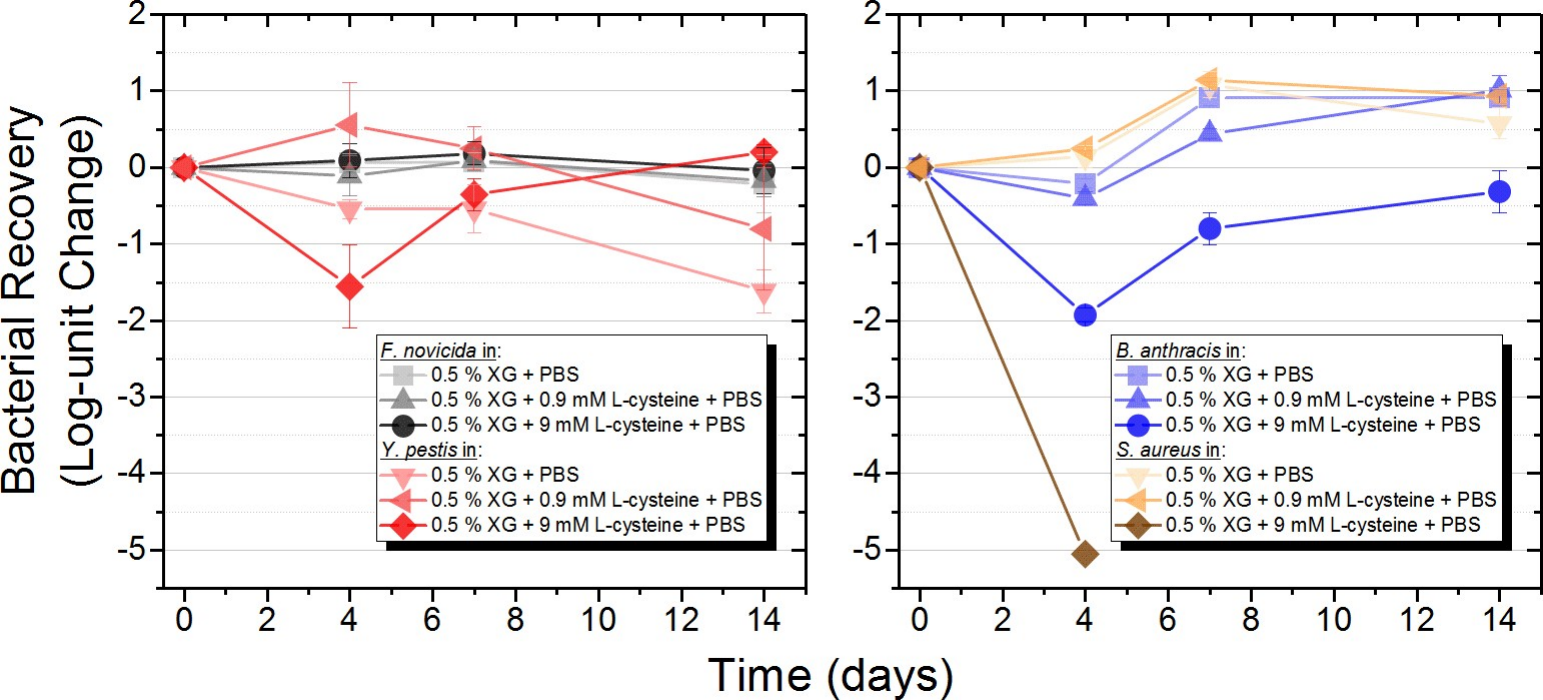
Stabilization of *B*. *anthracis*, *F*. *novicida*, *S*. *aureus*, and *Y*. *pestis* over time. Test matrices were spiked with ~10^5^ CFU/mL of Gram-negative *F*. *novicida* U112 or *Y*. *pestis* KIM D27 (pgm-) (left) or Gram-positive *B*. *anthracis* Sterne 34F2 or *S*. *aureus* ATCC 43300 (right). All were incubated at 22°C for 14 days. Viable plate counts were conducted at various time points to determine cell stability. Data are the average of three biological replicates and three sample replicates plated in triplicate (n = 9 measurements).

The addition of L-cysteine (9 mM) to XG in PBS improved viability preservation over time and at elevated temperatures for *F*. *novicida* U112. To determine the robustness of developed stabilization formulation, three additional organisms were added to the study. *Bacillus anthracis* Sterne, a Gram-positive spore-former, was selected because when placed under stress the cells sporulate and are inherently stable. Additionally, this organism is used as a surrogate for *Bacillus anthracis*, the causative agent of anthrax. A second Gram-positive organism, *Staphylococcus aureus* ATCC 43300, was selected because of clinical relevance and relative abundance in the built environment. *Yersinia pestis* KIM D27 was selected as a second Gram-negative model. Working towards a ‘universal’ stabilization formulation, the four bacterial strains were spiked into 0.5% XG in PBS, 0.5% XG in PBS with L-cysteine (9 mM), or 0.5% XG in PBS with L-cysteine (0.9 mM). Samples were stored at the more permissive temperature, 22°C, and viable plate counts were conducted over 14 days. Again, rather than working at a high concentration of cells, we targeted a lower concentration to mimic relevant environmental concentrations (~10^5^ CFU/mL). As expected, *F*. *novicida* was viable with little change in bacterial numbers in all three formulations (Fig 3, left), suggesting that L-cysteine is required only at higher temperatures. Similar trends were observed with the *Y*. *pestis* samples (Fig 3, left). As expected, total numbers of *B*. *anthracis* remained relatively similar but did increase slightly over time (Fig 3, right). Microscopic examination and differential heat counts revealed that the vegetative *B*. *anthracis* samples sporulated at day 4 and the majority of cells were in spore form. The average CFU/mL of the total number of cells (no heat treatment) was 9.9 × 10^4^where as the total number of spores following heat treatment was 5.7 × 10^4^. Solutions with XG in PBS with and without L-cysteine (0.9 mM) were able to maintain the starting cell concentration and are the preferred solutions to maintain *B*. *anthracis* viability over time. A ~2 log cell concentration decrease was observed after 4 days, followed by an increase over the next 10 days in the solution supplemented with L-cysteine (9 mM). Viable *S*. *aureus* cells were observed after 14 days when incubated in either 0.5% XG with L-cysteine (0.9 mM) or XG in PBS (~1 log growth) (Fig 3, right). Interestingly, no *S*. *aureus* cells were detected after 4 days in XG in PBS with L-cysteine (9 mM), demonstrating that the higher concentration of L-cysteine is detrimental to *S*. *aureus* cells. The use of other forms of cysteine as antimicrobials and anti-biofouling materials has been explored previously [19]. While the exact mechanism of inactivation needs to be further investigated, the observation that *S*. *aureus* cell numbers can be controlled by the concentration of L-cysteine could be beneficial if a sample is being collected from a high bio-burden location such as the built environment where *S*. *aureus* is prevalent [20]. In summary, 0.5% XG in PBS with L-cysteine (0.9 mM) was observed to be a ‘universal’ stabilization formulation for the four organisms tested at 22°C.

### Effect of storage temperature on viability of *F*. *novicida* and *S*. *aureus* in XG-based stabilization formulation

**Fig 4:**
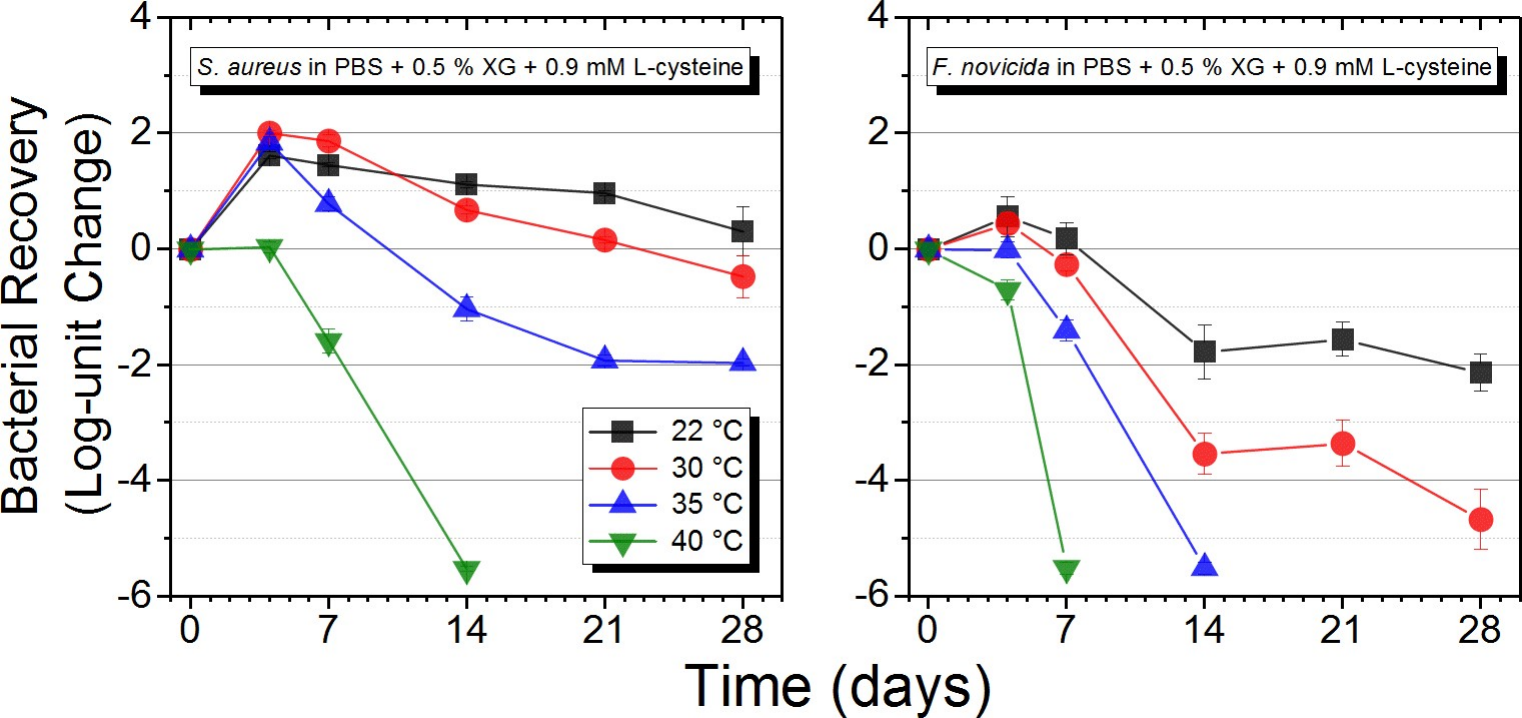
Role of temperature on *S*. *aureus* and *F*. *novicida* viability over time. Test matrices were spiked with ~10^5^ CFU/mL of *S*. *aureus* or *F*. *novicida* and incubated at 22–40°C for 28 days. Viable plate counts were conducted at various time points to determine cell stability. Data are the average of three biological replicates and three sample replicates plated in triplicate (n = 9 measurements).

Previous studies have demonstrated that increasing temperature can impact bacterial viability negatively, and this was further demonstrated in this study (Tables 2 and 3 and Fig 4). To further understand this observation, the effect of temperature on viability of *F*. *novicida* and *S*. *aureus* in 0.5% XG in PBS with L-cysteine (0.9 mM) was tested. Four temperatures relevant to a range of global storage temperatures (22, 30, 35, and 40°C) were tested, and viability was assessed via viable plate counts at days 0, 4, 7, 14, 21, and 28. The viability curves from the temperature experiments are provided in Fig 4. Regardless of organism, there was a clear correlation with decreased viability and increased storage temperature. Overall, fewer viable *F*. *novicida* cells (Fig 4, right) were observed across the examined temperatures compared to *S*. *aureus* cells (Fig 4, left). *F*. *novicida* cells were also more sensitive to heat than *S*. *aureus* cells, and viable cells were only recovered at 22 and 30°C after day 14. *S*. *aureus* cells remained viable in 0.5% XG with L-cysteine (0.9 mM) in PBS at 22, 30, and 35°C. No viable cells were recovered in samples incubated at 40°C after day 14 (Fig 4, right). These results suggest that temperature has an effect on maintaining viability in a sample, and a higher temperature shipment would not be recommended for shipment of biological samples in a transport media.

### Recovery and viability maintenance from cells deposited on surfaces

Preservation solutions must be compatible with collection methods; therefore, the stabilization solution was tested for the effectiveness on bacterial recovery from solid phase materials. *F*. *novicida*, *Y*. *pestis*, or *S*. *aureus* (~10^4^ CFU) were spiked onto stainless steel coupons and allowed to air dry for 2 hours. Cells were recovered using a macrofoam swab and the CDC swab method [11] prior to transfer into 0.5% XG in PBS with L-cysteine (0.9 mM). No viable *F*. *novicida* and *Y*. *pestis* cells were recovered immediately after collection, indicating that the cells did not survive the drying process. Viable *S*. *aureus* cells were recovered and remained viable for up to 28 days at 22°C after collection. The total number of cells recovered at day 0 was ~7 × 10^3^ CFU/mL, and the final concentration on day 28 was 1 × 10^5^ CFU/mL. This work shows viable *S*. *aureus* cells survive the drying process and can be stored in the stabilization formulation. Additional studies should be conducted on the effect of recovery of organisms from the coupons before viability can be tested.

### Nucleic acid stability in stabilization formulations

Nucleic acid stability was measured using real-time q-PCR. Samples were collected at various time points and then stored at −20°C so that all samples could be run at the same time. Samples were added directly to the PCR reaction to avoid DNA extraction steps. As previously reported, DNA is stable and detectable over a range of study conditions [4, 8]. The work here is in agreement with previous studies; DNA was detectable for the duration of the experiment, and concentrations were relatively unchanged for *F*. *novicida* and *S*. *aureus* across 14 days at 22 and 40°C (Table 4). The work presented here and by others demonstrates that maintaining bacterial viability is more challenging than stabilizing nucleic acids.

**Table 4:** Nucleic acid stability as assessed by real-time qPCR.

		***F*. *novicida*, Average C**_**T**_ **(standard deviation)**	
**Buffer Medium**	**Temperature**	Initial	Final (14 days)
**PBS**	22°C	25.97 (0.15)	26.99 (0.39)
	40°C	26.03 (0.37)	27.61 (0.57)
**0.5% XG in PBS**	22°C	26.47 (0.11)	26.44 (0.13)
	40°C	26.31 (0.20)	26.23 (0.13)
		***S*. *aureus*, Average C**_**T**_ **(standard deviation)**	
		Initial	Final (45 days)
**PBS**	22°C	26.91 (0.21)	21.22 (0.06)
	40°C	26.71 (0.09)	20.62 (0.07)
**0.5% XG in PBS**	22°C	27.97 (0.22)	29.13 (0.19)
	40°C	28.23 (0.12)	30.07 (0.52)

### Proteomic analysis of *F*. *novicida* stabilized in PBS and XG in PBS with L-cysteine

A proteomics study was performed to determine compatibility of the stabilization formulation with LC-MS/MS analysis and to identify protein changes of *F*. *novicida* in 0.5% XG in PBS with L-cysteine (0.9 mM) or PBS over time at 22°C. Cells remained viable when stored at 22°C for 45 days in the respective formulations (starting concentration ~10^7^ CFU/mL, final ~10^3^ CFU/mL). For proteomic analysis, triplicate biological cultures were grown. Each biological sample was then analyzed by LC-MS/MS in triplicate.

To identify peptides (and ultimately proteins) in these samples, data from *F*. *novicida* LC-MS/MS analyses were searched against the *F*. *novicida* U112 proteome sequences using the MSGF+ search algorithm. Peptide matches were filtered to yield a low false discovery rate (~0.01%), and protein identifications were made from peptide matches. Data are reported as protein spectral counts, which is a relative measure of protein abundance (S1 Table). While the samples stabilized in PBS yielded rich proteomic datasets with many *F*. *novicida* protein identifications, it was readily clear that there were significantly fewer *F*. *novicida* peptide/protein identifications in samples stabilized in XG.

After ruling out the possibility that different concentrations of peptide were loaded onto the LC column for XG samples, we hypothesized that the production process for XG likely leaves residual *Xanthomonas campestris* cellular proteins in xanthan gum. These *X*. *campestris* proteins may be present in XG-stabilized samples and thus are detected during analysis due to carryover or adherence to the bacterial cells. To determine if this was correct, MSGF+ searches were re-run and included both *F*. *novicida* and *X*. *campestris* protein sequences. Supplemental Fig 1 shows the overall abundance of *F*. *novicida* and *X*. *campestris* proteins identified in the PBS samples compared to the XG samples. Four hundred forty-eight *F*. *novicida* proteins were detected in PBS-stabilized samples (S1 Fig), but only 260 of those proteins were detected in XG-stabilized samples (S1 Fig). As hypothesized, there were abundant *X*. *campestris* proteins identified in the XG-stabilized samples (401 proteins; S1 Fig) and zero *X*. *campestris* proteins identified in the PBS samples (S1 Fig). The presence of *X*. *campestris* peptides in XG-stabilized samples interferes with detection of target peptides (i.e. *F*. *novicida* peptides) and thus restricts the ability to analyze protein expression changes of the organism of interest in this condition. Due to the high abundance of non-target proteins in XG, we have concluded that XG is not compatible with LC-MS/MS protein analysis. Further, we hypothesize that any biopolymers of biological origin or stabilizers may not be compatible with proteomics without extensive purification steps.

## Conclusions

In this study, we have demonstrated that bacterial viability and nucleic acid integrity can be maintained in xanthan gum formulations supplemented with L-cysteine to a higher extent than commercial reagents and buffers. Viable *B*. *anthracis*, *F*. *novicida*, *S*. *aureus*, and *Y*. *pestis* cells, as assessed via viable plate counts, were recovered from samples stored at 22, 30, 35, and 40°C up to 28 days. Not unexpectedly, *B*. *anthracis* formed spores in the formulations. Overall, bacterial viability declined with an increase in storage temperature, and additional studies are needed to improve thermostability. The results presented here provide an alternative storage and transport media for various microorganisms without a cold chain.

Addition of 9 mM L-cysteine, as compared to 0.9 mM L-cysteine, resulted in the rapid death of *S*. *aureus*. This finding could be crucial in controlling overgrowth of non-target *Staphylococcus* inadvertently collected from the built environment. Additional reagents such as Fungizone could be also used to control growth of non-target microbial populations. In summary, we demonstrated that polysaccharide-based formulations, specifically XG with L-cysteine, maintain bacterial viability and nucleic acid integrity across ambient and elevated temperatures for an array of both Gram-negative and Gram-positive bacteria.

## Supporting information

S1 Table Tabulated protein abundance data from the proteomics experiments. (XLSX)

S1 Fig Heat map showing *F*. *novicida* proteins and *X*. *campestris* proteins identified in PBS- and XG-stabilized samples. The total number of identified proteins from each organism was comparable (401 *X*. *campestris* proteins in XG and 448 in PBS *F*. *novicida* proteins). This demonstrates that xanthan gum contains a significant amount of *X*. *campestris* protein material. Samples are represented along the x-axis (columns), and proteins are shown on the y-axis (rows). Protein spectral counts (relative abundance) are represented by the color scale, with red representing more abundant, blue less abundant, and gray indicating the protein was not observed.
(DOCX)
