# Supplementary material for: Polysaccharide-based liquid storage and transport media for non-refrigerated preservation of bacterial pathogens
Source: PLoS One. 2019 Sep 6;14(9):e0221831. doi: 10.1371/journal.pone.0221831 (PMC6730858; doi:10.1371/journal.pone.0221831)
Supplement: S1 Fig — The total number of identified proteins from each organism was comparable (401 X. campestris proteins in XG and 448 in PBS F. novicida proteins). This demonstrates that xanthan gum contains a significant amount of X. campestris protein material. Samples are represented along the x-axis (columns), and proteins are shown on the y-axis (rows). Protein spectral counts (relative abundance) are represented by the color scale, with red representing more abundant, blue less abundant, and gray indicating the protein was not observed. (DOCX) [file pone.0221831.s002.docx]

Distribution Statement: Approved for public release; distribution is unlimited


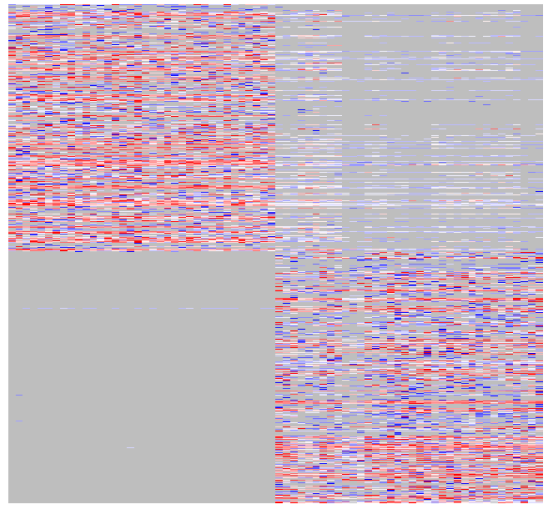

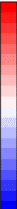


Less abundant

More abundant

Samples

XG samples

PBS samples

*X. campestris* proteins

*F. novicida* proteins

**Fig. S1. Heat map showing *F. novicida* proteins and *X. campestris* proteins identified in PBS- and XG-stabilized samples**. The total number of identified proteins from each organism was comparable (401 *X. campestris* proteins in XG and 448 in PBS *F. novicida* proteins). This demonstrates that xanthan gum contains a significant amount of *X. campestris* protein material. Samples are represented along the x-axis (columns), and proteins are shown on the y-axis (rows). Protein spectral counts (relative abundance) are represented by the color scale, with red representing more abundant, blue less abundant, and gray indicating the protein was not observed.
